# Interaction between Pirenzepine and Ninjinto, a Traditional Japanese Herbal Medicine, on the Plasma Gut-Regulated Peptide Levels in Humans

**DOI:** 10.1155/2013/907850

**Published:** 2013-03-27

**Authors:** Yuhki Sato, Itoh Hiroki, Yosuke Suzuki, Ryosuke Tatsuta, Masaharu Takeyama

**Affiliations:** Department of Pharmacy, Oita University Hospital, 1-1 Idaigaoka, Hasama-Machi, Oita 879-5593, Japan

## Abstract

The Japanese herbal medicine (Kampo) Ninjinto has been used for the treatment of gastroenteritis, esogastritis, gastric atony, gastrectasis, vomiting, and anorexia. The pharmacological effects of Ninjinto on the gastrointestine are due to changes in the levels of gut-regulated peptide, such as motilin, somatostatin, calcitonin gene-related peptide (CGRP), substance P, and vasoactive intestinal polypeptide (VIP). The release of these peptides is controlled by acetylcholine (ACh) from the preganglionic fibers of the parasympathetic nerve. Thus, we examined the effects of the selective M_1_ muscarinic receptor antagonist pirenzepine on the elevation of Ninjinto-induced plasma the area under the plasma gut-regulated peptide concentration-time curve from 0 to 240 min (AUC_0→240 min_) in humans. Oral pretreatment with pirenzepine significantly reduced the Ninjinto-induced elevation of plasma motilin and substance P release (AUC_0→240 min_). Combined treatment with Ninjinto and pirenzepine significantly increased the release of plasma somatostatin (AUC_0→240 min_) compared with administration of Ninjinto alone or placebo. Ninjinto appeared to induce the release of substance P and motilin into plasma mainly through the activation of M_1_ muscarinic receptors, and pirenzepine may affect the pharmacologic action of Ninjinto by the elevation of plasma substance P, motilin, and somatostatin.

## 1. Introduction

Kampo medicines, traditional herbal medicines, have been employed for a few thousand years and have contributed greatly to the treatment of many subjective symptoms. Kampo medicines are frequently prescribed with other synthetic or biotechnological drugs for the treatment of various chronic diseases. Such herbal medicines have been used in China for thousands of years and are now being manufactured in Japan as drugs. Most Kampo medicines are prepared from multiple crude herbs and contain many unrefined substances, and to determine the precise mechanism of the pharmacological effects of these medicines is too difficult. Ninjinto, a traditional Japanese Kampo medicine, is prepared from four different Chinese crude drugs. This medicine is one of the major prescriptions most frequently used for treatment of gastroenteritis, esogastritis, gastric atony, gastrectasis, vomiting, and anorexia in Japan. Al previous study has shown that Ninjinto not only significantly improved gastrointestinal motility but also showed stronger effects than those of some prokinetic drugs such as cisapride and metoclopramide in a rat model of postoperative ileus [1]. Hence, Ninjinto may be an effective herbal medicine for postoperative ileus.

One of the factors regulating gastrointestinal function has been suggested to be induction of changes in plasma levels of peptides such as motilin, somatostatin, calcitonin gene-related peptide (CGRP), and substance P. In recent years, some Kampo medicines of experimental gastrointestinal effects have been elucidated pharmacologically from the viewpoint of gut-regulated peptide levels. Among these reports, Naito et al. [2] previously confirmed that Ninjinto increased the levels of motilin, a powerful inducer of gastrointestinal motor activity and somatostatin, and that it participates in the control of gut motility by exerting both inhibitory and stimulating influences in human plasma. We also reported that Ninjinto increased the levels of cytoprotective peptides, CGRP, and substance P [3]. These results indicate that the action of Ninjinto is closely related to changes in motilin, somatostatin, CGRP, and substance P levels in plasma. 

Nervous mechanisms are important for the regulation of gastrointestinal motility and blood flow. A number of gut-regulated peptides such as CGRP and substance P have been localized immunohistochemically in nerves innervating the gastrointestinal tract [4]. The release of these peptides is controlled by acetylcholine (ACh) from the preganglionic fibers of the parasympathetic nerve [5–7]. Muscarinic receptors are also present on the membrane of motilin-secreting cells, and ACh is a major regulator of motilin release in the gastrointestine [8–11].

Pirenzepine, an antagonist specific for muscarinic M_1_ receptors, suppresses acid output and is used to treat gastritis and peptic ulcers clinically [12]. It has also been shown that this drug suppresses the elevation of serum gastrin levels by acting directly or indirectly on gastrin cells (G cells) [13, 14]. In clinical therapy, pirenzepine is often coadministered with Ninjinto. It is therefore of interest to examine the effects of pirenzepine on pharmacological action of Ninjinto release from the viewpoint of plasma gut-regulated peptide levels. 

The purpose of this study was to examine the effects of pirenzepine on the area under the plasma concentration-time curve from 0 to 240 min (AUC_0→240 min_) of Ninjinto-induced elevation of plasma CGRP-, substance-P-, motilin-, somatostatin-, and vasoactive-intestinal-polypeptides- (VIPs) immunoreactive substance (IS) in humans.

## 2. Materials and Methods

### 2.1. Materials

Ninjinto (EK-32, lot 01BJ), prepared as a 2.8 g dried powder extract in the proportion *Ginseng radix* (3.0 g), *Glycyrrhizae radix* (3.0 g), *Atractylodis rhizoma* (3.0 g), *Zingiberis siccatum rhizoma* (3.0 g), was kindly supplied by Kanebo Co. Ltd. (Tokyo, Japan). Pirenzepine (Gastrozepine tablet) was purchased from Boehringer Ingelheim Co. Ltd. (Hyogo, Japan). The VIP fragment peptide was supplied by Professor H. Yajima (Kyoto University, Kyoto, Japan). Antisera to VIP (A6054/R1B) and CGRP (CA1132) were purchased from Biogenesis (Poole, UK). Antisera to somatostatin (RA-08-108) and substance P (RA-08-095) were purchased from Cambridge Research Biochemicals (Cambridge, UK), and antiserum to motilin (Y121) was purchased from the Yanaihara Institute (Shizuoka, Japan). All other reagents were of analytical grade from commercial sources.

### 2.2. Subjects

Five healthy male volunteers (nonsmokers), aged 26–29 (median 27) years, weighing 55–68 (median weight 62) kg, with no history of gastrointestinal or hepatobiliary disease, and not taking any regular medications, participated in this study. No volunteers had received any medication for 1 month before the study, and there were at least 3-month intervals between participation in other studies. The volunteers had participated in more than three similar studies. 

### 2.3. Ethics

Each subject received information about the scientific purpose of the study, which was approved by the Ethics Committee of Oita Medical University, and gave informed consent for participation.

### 2.4. Study Schedule

Ninjinto or placebo (crystalline cellulose and lactose) was administered orally alone or 60 min after oral administration of pirenzepine 75 mg as a single dose of 6.0 g with water. The dose of test drugs used in this study was the maximum daily dose used in clinical therapy. For the same volunteers, pirenzepine (75 mg) or placebo (lactose capsules) was administered orally with 100 mL water. Each study was carried out with an interval of four weeks. Venous blood samples (10 mL) from a forearm vein were taken for enzyme immunoassay (EIA) of the levels of gut-regulated peptide. Samples were taken before and at 20, 40, 60, 90, 120, 180, and 240 min after administration of the drugs. All volunteers ate lunch (11:30–12:00) and the study was carried out from 14:00 (120 min after lunch) to 18:00.

### 2.5. Preparation of Plasma Extracts

The blood samples were collected in a chilled tube containing aprotinin (500-kallikrein inhibitor units/mL) and ethylenediaminetetraacetic acid (EDTA) (1.2 mg/mL). After centrifugation, the plasma samples were diluted with 4% acetic acid (pH 4.0), loaded onto Sep-Pak C_18_ cartridges (Millipore Corp., Milford, USA), and washed with 4% acetic acid. The peptides in the plasma were eluted with 70% acetonitrile in 0.5% acetic acid (pH 4.0), lyophilized, and reconstituted to 100 *μ*L with the assay buffer and subjected to EIA. For the EIA system, plasma samples were concentrated fivefold with Sep-Pak C_18_ cartridges. The recovery of plasma CGRP-, substance-P-, VIP-, motilin-, and somatostatin-IS was >90% with this extraction procedure.

### 2.6. EIA Procedure of CGRP, Substance P, VIP, Motilin, and Somatostatin

EIAs for CGRP- [15], substance-P- [16], VIP- [17], motilin- [18], and somatostatin-IS [19] were performed as previously described, by a delayed-addition method [20]. Separation of bound and free antigens was performed on an anti-rabbit IgG-coated immunoplates. The fluorescent product 4-methylumbelliferone was measured with an MTP-100F microplate reader (Corona Electric, Ibaraki, Japan). Human somatostatin, porcine motilin, fragment VIP (11–28), human CGRP (8–37), and substance P were conjugated with *β*-_D_-galactosidase (Boehringer Mannheim, Mannheim, Germany) with* N*-(*ε*-maleimidocaproyloxy)-succimide according to the method of Kitagawa et al. [21]. The EIAs for somatostatin, motilin, VIP, CGRP, and substance P were specific and highly sensitive to detection limits of 0.10, 0.80, 1.00, 0.08, and 0.40 fmol/well, respectively.

### 2.7. Data Analysis

Total release of peptides was calculated as the areas under the plasma concentration-time curves (AUC_0→240 min_) using the trapezoidal method. All values are expressed as mean ± s.d. (pg/mL). Comparisons of plasma peptide levels among blood sampling times were analyzed using Friedmann's test followed by the Mann-Whitney *U* test. Value of *P* < 0.01 or *P* < 0.05 was considered to represent a statistically significant difference.

## 3. Results 

### 3.1. Changes of the Release of Plasma CGRP-, Substance-P-, Motilin-, Somatostatin-, and VIP-IS (AUC_0→240 min_) after Ninjinto, with or without Pirenzepine Pretreatment

The total release of plasma CGRP-, substance-P-, motilin-, somatostatin-, and VIP-IS (AUC_0→240 min_) after administration of Ninjinto, with or without pirenzepine pretreatment is shown in Table 1. The total release of CGRP (AUC_0→240 min_) increased significantly after administration of Ninjinto (7937.7 ± 2485.9 pg mL/min) compared with placebo (5187.0 ± 964.9 pg mL/min) but did not change after the administration of Ninjinto with pirenzepine pretreatment (7949.7 ± 2465.2 pg mL/min). The total release of substance P (AUC_0→240 min_) after administration of Ninjinto with pirenzepine pretreatment (4313.2 ± 1226.4 pg mL/min) significantly decreased compared with Ninjinto alone (9815.0 ± 2182.7 pg mL/min). The total release of motilin (AUC_0→240 min_) after administration of Ninjinto alone (28360.0 ± 3017.4 pg mL/min) significantly increased but decreased after administration of Ninjinto with pirenzepine pretreatment (11997.4 ± 2483.2 pg mL/min), compared with Ninjinto alone and placebo group (20481.0 ± 5693.6 pg mL/min). The total release of motilin (AUC_0→240 min_) decreased after the administration of Ninjinto with pirenzepine pre-treatment (11997.4 ± 2483.2 pg mL/min), compared with Ninjinto alone and placebo (28360.0 ± 3017.4 and 20481.0 ± 5963.6 pg mL/min, resp.). After the administration of Ninjinto with or without pirenzepine pretreatment, no significant changes were observed in the total release of VIP (AUC_0→240 min_).

### 3.2. Changes of the Release of Plasma CGRP-, Substance-P-, Motilin-, Somatostatin-, and VIP-IS Levels after Pirenzepine

The total releases of CGRP, substance-P-, motilin-, somatostatin-, and VIP (AUC_0→240 min_) after administration of pirenzepine are shown in Table 2. The total release of CGRP (AUC_0→240 min_) increased after administration of pirenzepine (8864.9 ± 1581.8 pg mL/min) compared with placebo (6306.6 ± 542.0 pg mL/min). After the administration of pirenzepine, no significant changes were observed in the total release of substance-P (AUC_0→240 min_). The total release of somatostatin (AUC_0→240 min_) significantly increased after administration of pirenzepine (4779.3 ± 858.5 pg mL/min) compared with placebo group (3483.8 ± 345.7 pg mL/min). After the administration of pirenzepine, no significant changes were observed in the total release of VIP (AUC_0→240 min_).

## 4. Discussion

In our study, Ninjinto significantly increased the release of plasma CGRP, substance P, motilin, and somatostatin. The coadministration of Ninjinto with pirenzepine pretreatment significantly decreased the release of plasma substance P and motilin and increased that of plasma somatostatin compared with Ninjinto alone or placebo.

CGRP is a powerful vasoactive substance that is released from the sensory afferent nerve endings in response to gastric mucosal injury in the stomach. CGRP increases gastric mucosal blood flow as a gastroprotective factor [22]. In addition, CGRP has a potent effect on gastrointestinal motility and secretion [23]. In our study, Ninjinto significantly raised the releases of plasma CGRP after administration to healthy volunteers, while Ninjinto with pirenzepine pretreatment had not changed compared with Ninjinto alone and placebo. Previous studies demonstrated that CGRP is localized in motor neurons and nerve terminals at the neuromuscular junction, where it coexists with ACh and that CGRP is a neurotransmitter that may act in conjunction with Ach [24]. Trasforini et al. [6] reported that the acetylcholinesterase inhibitor caused significant increases in basal plasma levels of CGRP in healthy humans, whereas pirenzepine failed to exert any significant effect. Thus, it was thought that the effects of Ninjinto on CGRP levels might be not related in M_1_ muscarinic receptor. 

Substance P coexists with CGRP in the sensory afferent neurons of the gastrointestinal mucosa and is released with ACh in response to depolarizing stimulation in the enteric nervous system [25]. In the gastrointestinal tract, substance P is thought to be involved in the control of motility, secretion, and blood flow [26]. In our study, Ninjinto raised the release of plasma substance P after administration to healthy volunteers, while that after the administration of Ninjinto with pirenzepine pretreatment caused decreases compared with Ninjinto alone or placebo. Schmidt and Holst [27] reported that muscarinic receptors are present on the intrinsic substance-P-producing neurons and inhibit the release of substance P in porcine ileum. Although it has not previously been confirmed whether M_1_ muscarinic receptors are present on the substance-P-producing neurons in gastrointestinal tract, these results indicate that the effects of Ninjinto on plasma substance P levels might be related to M_1_ muscarinic receptor.

Motilin is one of the most important factors controlling the regular occurrence of phase III of interdigestive migrating contractions [28]. Muscarinic receptors are present on the membrane of motilin-secreting cells, and ACh is a major regulator of motilin release by acting on M_1_ or M_3_ muscarinic receptors. In our results, the administration of Ninjinto with pirenzepine pretreatment decreased the release of plasma motilin compared with Ninjinto alone. Thus, it is thought that the effect of Ninjinto on the release of plasma motilin might be blocked by the administration of pirenzepine. Also, it is known that somatostatin inhibits the secretion of motilin in gut [29]. Pirenzepine-elevated somatostatin might be closely related to part of decreases in plasma motilin levels. Our findings suggest that Ninjinto may stimulate motilin cells mainly *via* M_1_ muscarinic receptors.

Somatostatin is a gastrointestinal motility stimulator and functions as an inhibitor of motilin and gastrin release in digestive organs [30]. In our results, the administration of Ninjinto with pirenzepine pretreatment significantly increased the release of plasma somatostatin compared with placebo. Sue et al. [14] reported that pirenzepine acts on somatostatin cells (D cells) to promote somatostatin release, thereby indirectly suppressing gastrin release from G cells in the stomach. In our study, pirenzepine significantly increased the release of plasma somatostatin. These results show that the effects of Ninjinto with pirenzepine pretreatment on plasma somatostatin may be related to direct or indirect action by Ninjinto and pirenzepine. 

VIP has a vasodilating effect and is a gastrointestinal motility regulator. VIP is considered to be one of the neurotransmitters released from nonadrenergic, noncholinergic inhibitory neurons in the gastrointestinal tract [31]. We found that Ninjinto with or without pirenzepine pretreatment had no effect on plasma VIP-IS levels. 

According to the report by Sekiya and Morimoto [1], Ninjinto significantly might increase plasma gut-regulated peptide levels in the treatment of diseases of the ileus and its changes might contribute to promoting gastrointestinal motility. Therefore, the pharmacological action of Ninjinto might be partially due to the changes in plasma gut-regulated peptides. In our results, the coadministration of Ninjinto with pirenzepine pretreatment caused a significant decrease in plasma substance P, motilin, and somatostatin releases, compared with Ninjinto alone or placebo. It was estimated that the use of pirenzepine might affect the clinical efficacy of Ninjinto, although it is certain whether these effects in healthy volunteers are the same as those in patients clinically. Therefore, it is necessary to investigate the effects in patients with a condition such as ileus.

## 5. Conclusions 

Ninjinto might increase the release of plasma substance P and motilin *via* M_1_ muscarinic receptors. Coadministration of Ninjinto with pirenzepine decreases Ninjinto-induced substance P and motilin release and increases that of somatostatin release. But CGRP and VIP have no changes. Although further studies are needed, our findings may be important clinically.

## Figures and Tables

**Table 1:** Total amount of substance-P-, CGRP-, and VIP-IS released in plasma after the administration of Ninjinto (6.0 g) to 5 healthy volunteers with or without pirenzepine (75 mg) treatment.

	Plasma AUC_0→240__min_ (pg mL/min)		
	Ninjinto (6.0 g)	Ninjinto (6.0 g) + pirenzepine (75 mg)	Placebo
CGRP	7937.7 ± 2485.9*	7497.7 ± 2465.2	5187.0 ± 964.9
Substance P	9815.0 ± 2182.7	4313.2 ± 1226.4^†^	5104.5 ± 1091.1
Motilin	28360.0 ± 3017.4	11997.4 ± 2483.2^∗∗†^	20481.0 ± 5693.6
Somatostatin	2870.0 ± 512.1*	3016.2 ± 462.1**	1928.7 ± 122.9
VIP	2005.9 ± 793.4	2004.2 ± 915.1	2011.2 ± 298.7

Each value represents the mean ± s.d. *n* = 5. AUC_0→240__min_, area under the plasma concentration-time curve from 0 to 240 min. *P < 0.05 and **P < 0.01 significantly different compared with placebo. ^†^ *P* < 0.05 significantly different compared with Ninjinto alone.

**Table 2:** Total amount of CGRP-, substance-P-, motilin-, somatostatin-, and VIP-IS released in plasma after the administration of pirenzepine (75 mg) to 5 healthy volunteers.

	Plasma AUC_0→240__min_ (pg mL/min)	
	Pirenzepine (75 mg)	Placebo
CGRP	8864.9 ± 1581.8**	6306.6 ± 542.0
Substance P	2979.4 ± 1350.0	5104.5 ± 1091.1
Motilin	14010.7 ± 9483.4	20481.0 ± 5693.6
Somatostatin	4779.3 ± 858.5*	3483.8 ± 345.7
VIP	1645.6 ± 551.5	2011.2 ± 298.7

Each value represents the mean ± s.d. *n* = 5. AUC_0→240__min_, area under the plasma concentration-time curve from 0 to 240 min. *P < 0.05 and **P < 0.01 significantly different compared with placebo.
